# Employing discrete global grid systems for reproducible data obfuscation

**DOI:** 10.1038/s41597-024-03354-5

**Published:** 2024-05-17

**Authors:** Gino Caspari, João dos Santos Manuel, Ana Gago-Silva, Michael Jendryke

**Affiliations:** 1 Max Planck Institute of Geoanthropology, Kahlaische Str. 10, 07745 Jena, Germany; 2 Institute of Archaeological Sciences, University of Bern, Mittelstrasse 43, 3012 Bern, Switzerland; 3 GeoInsight AG, Forstweg 65A, 3012 Bern, Switzerland

**Keywords:** Archaeology, Geography

## Abstract

Archaeological heritage worldwide is threatened through deliberate destruction in particular site looting making the location of archaeological sites potentially sensitive data. At the same time, public information about site locations are important for heritage management, as well as agricultural and urban development. Finding a balance between revealing detailed site locations and not providing data at all is a difficult task. Here we provide an approach to obfuscate archaeological site location data facilitated through a Discrete Global Grid System. We then apply the new obfuscation method to the global p3k14c data set. Veiling the locations of heritage sites with a Discrete Global Grid System allows tiered accuracy access for different stakeholders tailored to their respective needs as well as legal constraints and requirements of administrations. Discrete Global Grid System based obfuscation is globally scalable, consistent, reproducible, and can address the current heterogeneity of obfuscation methods.

## Introduction

Archaeological sites worldwide are under threat of looting^1^. The illicit excavation of archaeological artifacts and the destruction of related archaeological contexts is detrimental to the preservation of important heritage sites and archaeological knowledge production. Once archaeological artifacts are removed from context, a large portion of the potentially recoverable information is irretrievably lost. Long-term, entire categories of archaeological artifacts become disassociated from their respective contexts. Famously, this is the case with Cycladic marble figurines for which it has become impossible to reliably reconstruct their function, chronological sequences, and regional variations^2^. While recent years have seen an increased interest in the topic from an academic point of view, protection, especially for sites in remote locations, is still in its infancy in practical terms. Methods to detect looting from remote sensing data have been developed^3–9^ but their application remains geographically spotty and is rarely applied beyond local case studies as a practical management tool. The direct threat of looting thus makes location data of archaeological sites potentially sensitive.

Coordinate obfuscation is more generally used in data privacy to protect sensitive location information while still enabling the utilization of spatial data for analysis and research purposes. By introducing intentional changes to geographic coordinates, obfuscation preserves data utility and creates a chosen level of confidentiality. The potentially problematic nature of the availability of high-resolution location information is especially apparent when it comes to the privacy of individuals where a plethora of legal safeguards are in place to preserve confidentiality. Scientific fields from epidemiology to logistics and transport planning have long applied obfuscation methods in order to protect individual privacy. Coordinate obfuscation methods are trying to find a solution to privacy or security concerns by balancing data utility and confidentiality. In epidemiological studies, spatial data are likely to contain sensitive personal information. Applying coordinate obfuscation protects the study subject’s privacy while still allowing scientists to process the data for disease patterns, identifying e.g. high risk areas or providing policy advice on how to optimize public health resource allocation [cf.^10,11^]. Coordinate obfuscation finds application in transportation planning, where travel patterns of individuals are crucial for infrastructure development and traffic management [cf.^12,13^]. By obfuscating location data, transportation planners can preserve individual privacy while assessing traffic flows, identifying congestion hotspots, and improving transportation systems. In environmental research, spatial data that include sensitive information about endangered species or protected habitats are regularly obfuscated^14^. Coordinate obfuscation allows scientists to share aggregated or perturbed data, ensuring the preservation of ecological privacy. In this way the impacts of human activities on the environment can be studied while safeguarding important locations. Urban planning and public safety initiatives often require detailed location information. However, revealing exact coordinates may compromise the privacy and security of individuals. Coordinate obfuscation enables city planners and law enforcement agencies to analyze crime patterns, optimize emergency response routes, and design safer neighborhoods while maintaining the anonymity of residents and victims [cf.^15^].

Archaeologists have recognized that there is a trade-off between making data available for research and cultural heritage management purposes on one side and the need for archaeological site protection on the other. In some cases, this is underpinned by national legislation that has been passed to further the protection of cultural heritage such as Section 9 of the US Archaeological Resources Protection Act of 1979 which explicitly states that “information concerning the nature and *location* of any archaeological resource for which the excavation or removal requires a permit or other permission under this Act or under any other provision of Federal law may not be made available to the public […]”^16^ [emphasis by the authors]. This requires tools to obfuscate the coordinates of archaeological sites while maintaining a level of usability for scientific analytical purposes. Large archaeological data bases thus inevitably have to address this question.

The SahulArch data base for example approached the problem through applying a randomizing algorithm to the data and storing them as polygons^17^. The p3k14c data base, a global database of radiocarbon dates, maintains legal compliance through obfuscating the coordinates of archaeological sites in the United States and Canada by transforming them into the centroid of the administrative subdivision in which the data point is located^18^. They, however, only perform obfuscation for North American sites. The Digital Archaeological Record (tDAR) obfuscates sites which are under one square mile in size and displays larger sites in their actual extent^19^. The Digital Index of North American Archaeology (DINAA) uses county or park centroids to indicate site location^20^. The Central Asian Archaeological Landscapes Project solves the issue through providing site locations open access at a lower map resolution down to c. 1:320,000 and requires users to register in order to obtain more accurate data^21^. How archaeological site obfuscation is approached varies from project to project. As there is a clear need for coordinate obfuscation solutions but little guidance on how to accomplish it in a consistent manner. The landscape of archaeological site obfuscation is highly heterogeneous and this lack of standards is far from ideal.

In this article, we aim to introduce a new consistent method for coordinate obfuscation with which controlled perturbations to geographic coordinates can be implemented. These perturbations introduce a degree of fuzziness, preventing direct identification of specific data points while preserving the overall spatial patterns of the data.

## Results

**Fig. 1:**
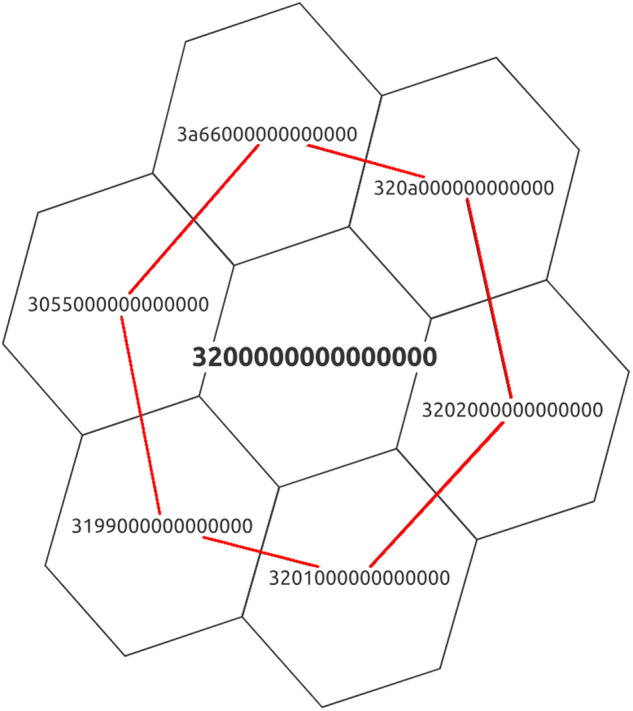
The principle of the DGGS with Aperture 3. The center point of a larger cell is also the center of a smaller cell, while the area of a larger cells is equivalent to the area of 3 smaller cells, hence aperture 3.

We use a Discrete Global Grid System to conceal the exact coordinates of cultural heritage sites. Our chosen DGGS is an Icosahedral Snyder Equal Area aperture 3 Hexagon (ISEA3H) Geodesic DGGS^22^, which divides the Earth into hexagonal regions of similar size and number of neighbors. Aperture 3, as shown in Fig. 1, means that the area of one larger cell (*3200000000000000*) equals the areal size of three smaller cells of the immediate next higher resolution and ensures consistency when calculating spatial relationships. The chosen DGGS is a hierarchical system where each hexagon has exactly six neighbors and seven children (with the exception of the 12 pentagons created due to the underlying icosahedron), where the ID of the child cell, directly under the parent, is identical to the parent ID but with a different resolution/security level. The input of a point returns the respective hexagon based on a specified security level (spatial resolution) between 1 and 36, as detailed in Table 1.

**Table 1:** Security level 1 to 36 with corresponding average cells size in km^2^.

Security Level	Number of cells (globally)	Area in km2
1	12	51006562.172408800
2	32	17002187.390802900
3	92	5667395.796934310
4	272	1889131.932311440
5	812	629710.644103813
6	2432	209903.548034604
7	7292	69967.849344868
8	21872	23322.616448289
9	65612	7774.205482763
10	196832	2591.401827588
11	590492	863.800609196
12	1771472	287.933536399
13	5314412	95.977845466
14	15943232	31.992615155
15	47829692	10.664205052
16	143489072	3.554735017
17	430467212	1.184911672
18	1291401632	0.394970557
19	3874204892	0.131656852
20	11622614672	0.043885617
21	34867844012	0.014628539
22	104603532032	0.004876180
23	313810596092	0.001625393
24	941431788272	0.000541798
25	2824295364812	0.000180599
26	8472886094432	0.000060200
27	25418658283292	0.000020067
28	76255974849872	0.000006689
29	228767924549612	0.000002230
30	686303773648832	0.000000743
31	2058911320946490	0.000000248
32	6176733962839470	0.000000083
33	18530201888518400	0.000000028
34	55590605665555200	0.000000009
35	166771816996665000	0.000000003
36	500315450989997000	0.000000001

**Fig. 2:**
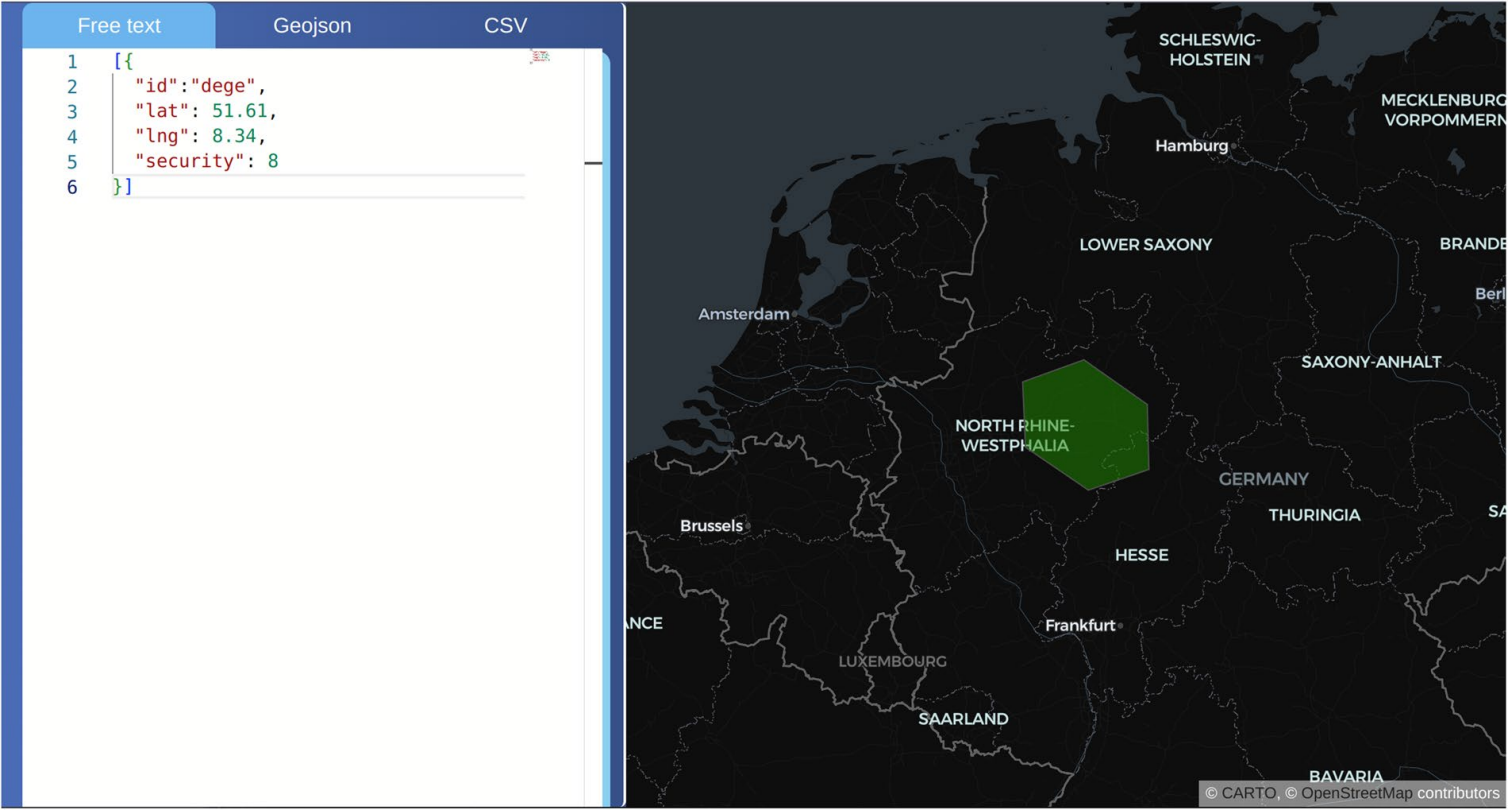
The web interface at obfuscate.geoinsight.ai.

To visually demonstrate the spatial obfuscation process, we have built an openly accessible web interface. For this purpose the technologies used were JavaScript (ReactJs), HTML, and CSS. The interface can be reached through obfuscate.geoinsight.ai. The interface has two sections (Fig. 2):

1. A web map, using a WGS84 projection, to show the DGGS grid cells and
2. A panel to convert a geographic coordinate pair or geometry into DGGS grid cells.

To visualize the grid cells, we use a map on the browser, implementing the open-source library deck.gl. Deck.gl is a WebGL-based visualization web tool for large-scale data sets^23^. The library provides a coordinate space to visualize 2D or 3D map data on the browser. For our intended purposes, the coordinate system used was the default one, WGS84, with latitude and longitude coordinates in degrees from Greenwich meridian/equator respectively, and altitude in meters above mean sea level. For aesthetic and guiding purposes, we used a global base map along deck.gl. The base map used is provided by Carto^24^.

A panel allows to input a set of coordinate pairs, or more complex geometries using the GeoJSON notation. GeoJSON serves as a geospatial data exchange format rooted in JavaScript Object Notation (JSON). It outlines various JSON object types and outlines the methodology for assembling these objects to convey information regarding geographic features, their attributes, and their geographical boundaries. GeoJSON adopts the World Geodetic System 1984 as its geographic coordinate reference system and measures units in decimal degrees^25^. The input box is a web Integrated Development Environment (IDE), i.e. a code editor. The process of obfuscating coordinates is implemented as follows:

1. The user types the input coordinates or geometries on the panel.
2. The coordinates are sent through an HTTP Application Programming Interface endpoint to the backend.
3. The intersection between the set of coordinates and the global grid is identified.
4. The endpoint then outputs a grid cell or set of grid cells which are then displayed on the map.

Every time the input is edited, the results will change on the map accordingly. The interface is designed to respond immediately, i.e. depending on the input inserted in the panel a response will always be given. If the input inserted is incorrect, an error message will be displayed. Errors can occur due to wrong syntax on the IDE, an endpoint response error, or a faulty endpoint output message. The error message will always be shown above the panel. If the input is correct, then the response will be shown on the map.

The conversion panel of the application encompasses three options: A free text solution that uses Java Script Object Notation (JSON), a GeoJSON file uploader or editor, and a CSV file uploader or editor. The free text option uses a customized input as a JSON, and only needs to send a pair of coordinates (latitude and longitude) or a set of pairs. It is mandatory that a JavaScript array of objects is sent, otherwise it will fail and a syntax error will be shown. In more detail, a JavaScript object is a code notation, which needs be enclosed by curled braces, and everything inside the object contains a set of pairs of property/value:

{

“property1”: “value1”,

“property2”: “value2”,

“property3”: “value3”

}

A JavaScript array stores a collection of multiple items, enclosed by brackets. These items can be a number, text, object, another array, boolean, undefined, or a null value:

[1, 2, “e”]

A JavaScript array of objects will then have the following notation. Example:

[

{

“key1”: “value1”,

“key2”: “value2”

},

{

“key1”: “value1”,

“key2”: “value2”,

},

]

For the current case, the input for the free text solution needs to follow a specific data model, every object will contain a latitude/longitude coordinate point, a resolution grid level, and an id. These properties are described in Table 2:

**Table 2:** Properties of free text input for coordinate obfuscation.

Properties	Description
id	The identifier for each point
lat	Latitude for the point. Values range from −90 to 90
lon	Longitude for the point. Values range from −180 to 180
security	Grid resolution level. Integer values range from 1 to 36.

The GeoJSON option has two sub-options, an input box much alike the free text, and a GeoJSON file uploading widget. This option also follows the same code notation like the free text, where it uses a JSON object, but in this case it follows a specific format. The GeoJSON format is an open source specification that allows the encoding of a number of geographic data structures. There are other geometry types in the GeoJSON format, but input box so far only accepts type “point”. If the user uses the file loader, then the file extension needs to be on a.geojson or.json. The process is similar to the previous option, every time the user edits or types in the input box, the output on the map changes. The same happens, if the user changes the file. The CSV option behaves similarly to the GeoJSON option, with the two sub-options, an input box, and a CSV file uploading widget. The changes on these inputs will be reflected on the map, the difference here is that the code notation is different and the extension used is .csv. The following Table 3 describes the inputs used for each option:

**Table 3:** Input formats for the free text, GeoJSON, and CSV input options.

Free text	GeoJSON	CSV
[{ “id”:“dege”, “lat”: 7, “lng”: −32, “security”: 13 }]	{ “type”: “FeatureCollection”, “features”: [{ “type”: “Feature”, “properties”: { “id”: “defe”, “security”: 2 }, “geometry”: { “type”: “Point”, “coordinates”: [ 40.40625, 16.29905101458183] } }] }	id,lat,lng,security id0,47, −32,2 id0,45, −65,14

## Discussion

We use the p3k14c data set to illustrate the utility of the obfuscation method on a global scale. The p3k14c database is a collection of 180,070 radiocarbon dates from archaeological site locations worldwide. The data set incorporates data points from other databases and also includes information from scientific publications which have not been integrated into other databases. Due to the variations in radiocarbon measurement and reporting standards across different regions, the database applies a quality control. After manual cleaning and an automated scrubbing process, data for the United States and Canada as well as for the data set published by d’Alpoim Guedes & Bocinsky^26^ were then obfuscated “in order to protect site locations”^18^. The authors chose not to obfuscate other data points as they were already openly available^18^. The chosen obfuscation method introduces a degree of heterogeneity to the data set which reduces its usability for fine-grained analyses. While this might not matter for coarse global analyses, consistency of accuracy within the same data set has advantages for the investigation of spatial phenomena on different scales.

**Fig. 3:**
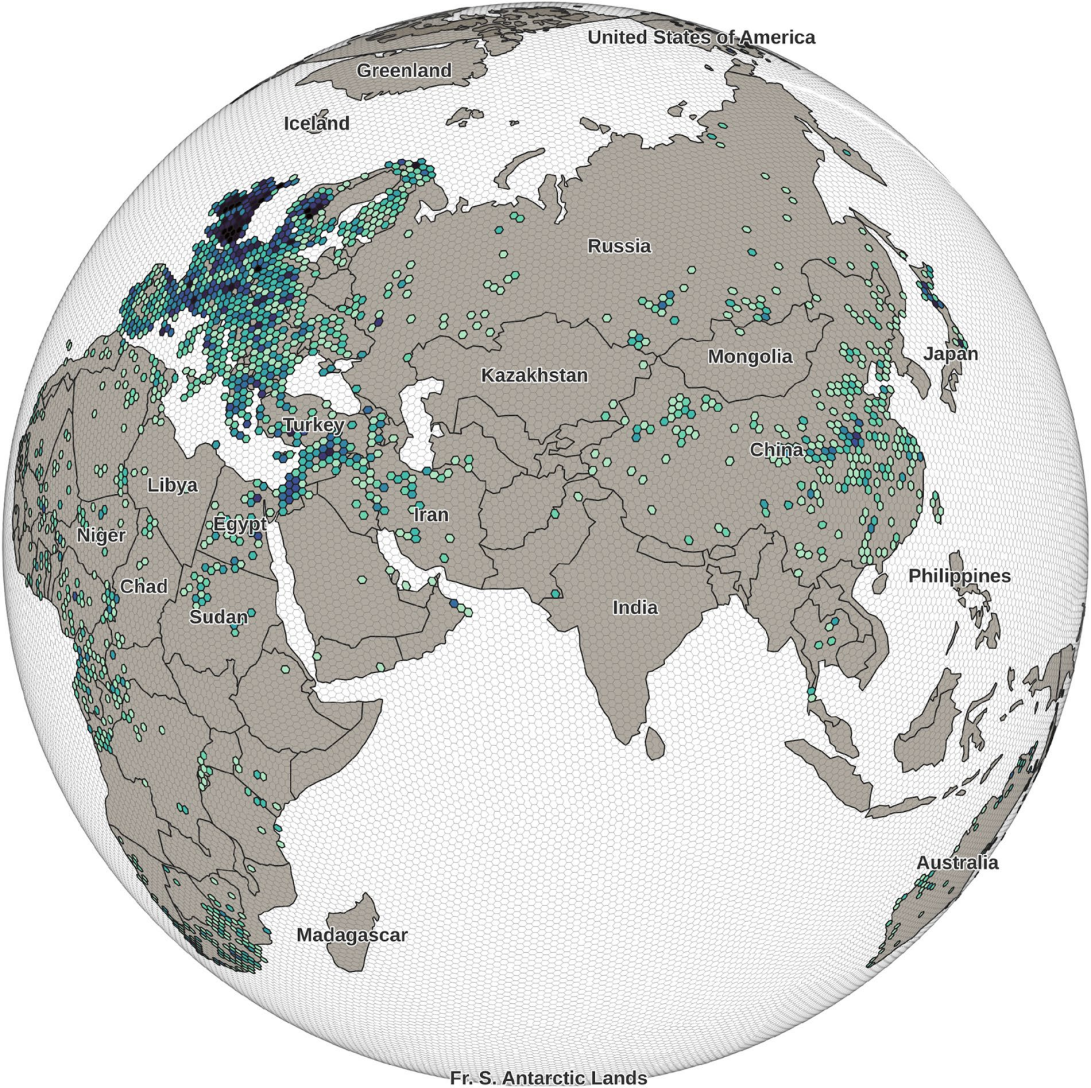
Globally obfuscated p3k14c data set at security level 8, colored according to the number of sites per cell for visualization.

Figure 3 shows the results of the obfuscation of the global p3k14c data set with security level 8. The densities of data points and the enormous spatial gaps in between are immediately apparent. Europe and the United Kingdom in particular are very densely populated with data while entire nation states in Sub-Saharan Africa, Central Asia and Oceania remain virtually empty. In this way the obfuscation method could also serve to quickly visually assess potential geographic biases and disparities in large point data sets.

**Fig. 4:**
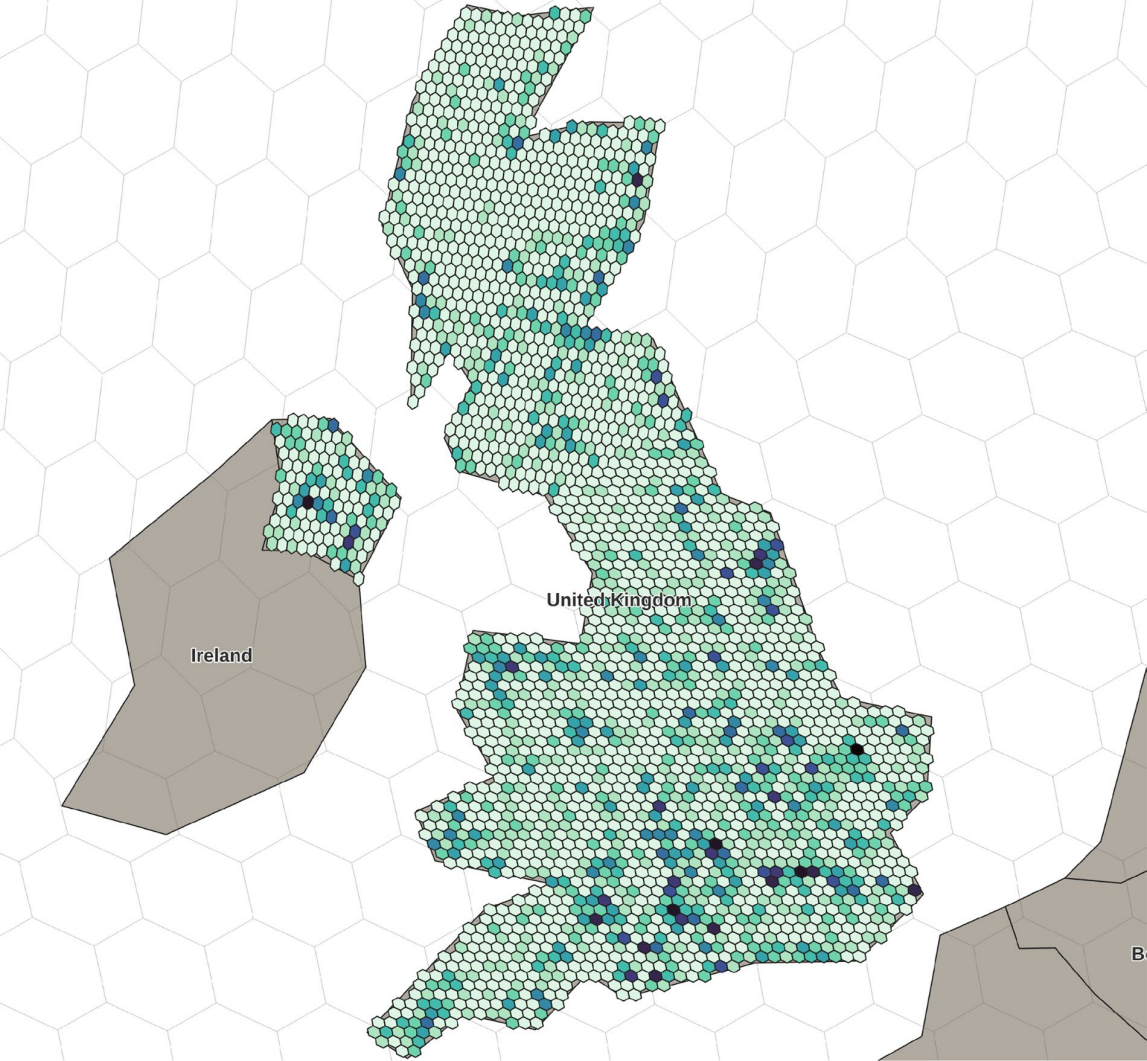
Obfuscated p3k14c United Kingdom data set at security 12, colored according to the number of sites per cell for visualization.

Zooming into the United Kingdom and using security level 12 (Fig. 4), we gain a better understanding of the geospatial distribution of data points at a national level. This can serve to identify hotspots of activity but also highlight gaps in the record. When applying this to national or regional archaeological heritage databases, the tool cannot only be used for the obfuscation of site locations but also allow for insights into the archaeological heritage record.

Crucially, the obfuscation method we introduce here is globally scalable and consistent. Due to the ISO-standardization of the used discrete global grid, the obfuscation is not based on an arbitrary obfuscation algorithm, curtailing of coordinates or other calculations which are based directly on the underlying data. The independence between obfuscation method and obfuscated data is key to providing a result which cannot be reverse engineered or otherwise approximated.

**Fig. 5:**
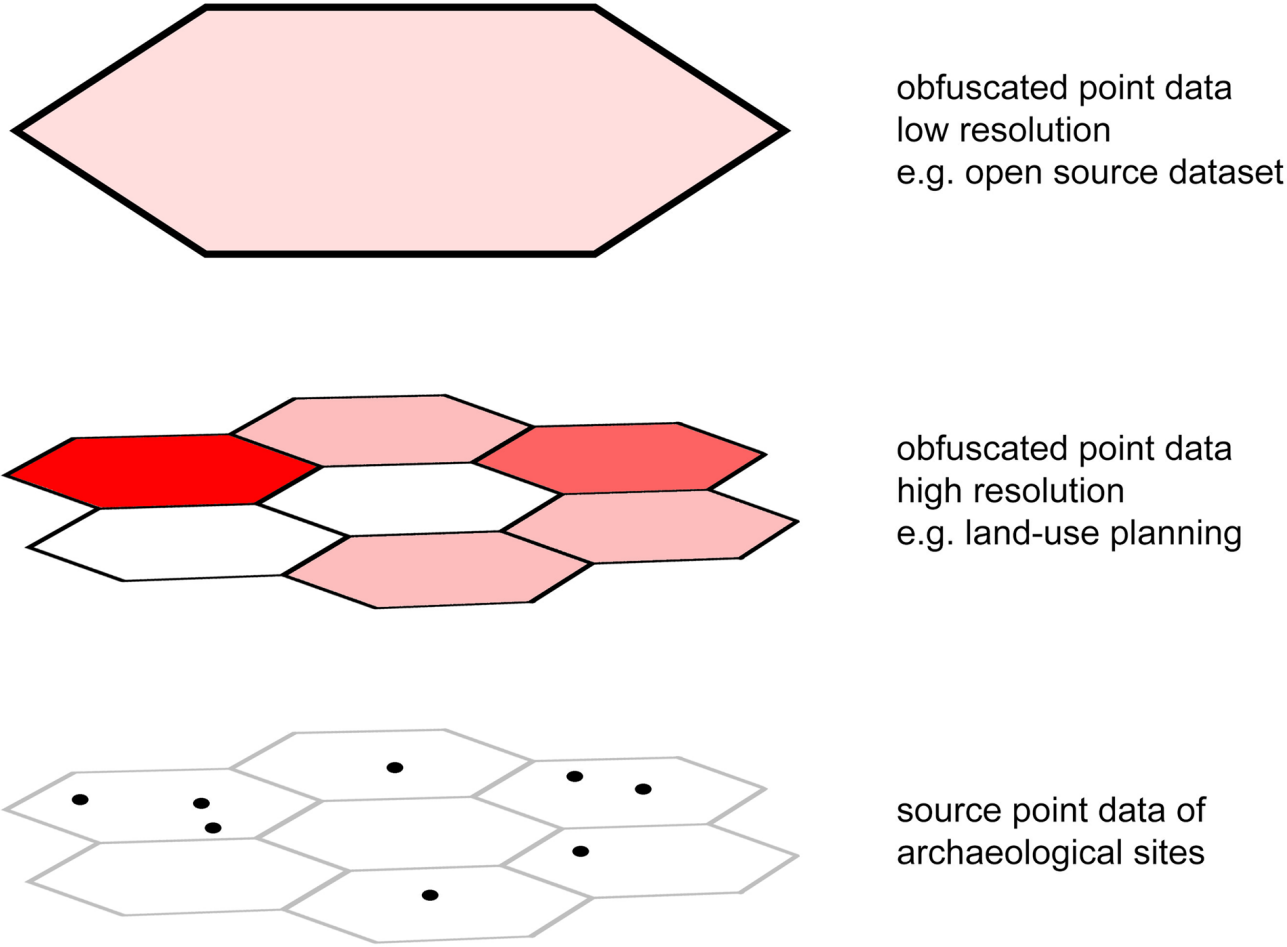
Multiple levels of obfuscation allow for tiered accuracy access to sensitive data.

The tiered accuracy access over 36 different levels can be used in order to grant different stakeholders different access (Fig. 5). While state archaeologists and cultural heritage managers will need high resolution data for management purposes this is not the case for the general public. In recent years a clear need to make heritage-related data accessible to the general public has emerged^15,27^. Making heritage data publicly available contributes to a reintegration of heritage knowledge into the broader community. This process has to happen in a balance between access and protection where there is always a trade-off between increasing the interaction with cultural heritage in all of its forms and safeguarding oftentimes unique and fragile cultural remains. Other administrative stakeholders like forest management services, urban and land-use planners, agricultural production and construction companies will have different needs regarding the resolution of location data of heritage sites and thus might be granted differently tiered access. We do not claim to understand these needs in particular detail and there are significant geographic differences based on diverse and changing local rules, regulations, and practices. We therefore merely suggest a standardized approach which is highly customizable while maintaining global transferability.

In this article we have provided a globally scalable, consistent, and ISO-standard derived method for the obfuscation of point data sets of archaeological heritage sites. We find that bringing consistency to the highly heterogeneous landscape of obfuscation methods in the heritage sector would be beneficial to researchers and administrators alike. The method’s widespread adoption would facilitate data integration, interoperability, and transferability, allowing for scalable global models within the field of archaeological and heritage research. The introduced approach offers a customizable tiered accuracy access framework to archaeological heritage managers in accordance with local laws. In this way, archaeological heritage sites can be protected while information is made available to different stakeholders based on their respective needs. Discrete Global Grid Systems provide a way to consistently and reproducibly obfuscate any kind of coordinates.

## Methods

There are various ways in which an archaeological site can be represented in a geographic information system. The most basic and widespread representation is in the form of a point, ideally the center point of the site, declared by a coordinate pair for latitude and longitude. Alternatively, a site’s location may be identified by the border line around the site or as a polygon covering the site’s area. Finally, a site’s location could just be a toponym: the name of a valley, mountain or other geographic feature, without measurable values to determine the exact location. A site’s location must be expressed through coordinates in decimal degrees (WGS84), where we ignore the z-axis and assume that this location is on the surface of the earth.

A lot of research has been conducted in relation to geo-obfusaction in the field of privacy, security, and location based services. The findings can be transferred and applied to archaeology and heritage site management as well. While article titles of scientific contributions like “SpotME if you can:…”^28^ or “Track me if you can:…”^29^ indicate that obfuscation has been primarily about individual privacy, many methods can equally be applied to immobile objects and structures. Privacy preserving techniques discuss different forms of location obfuscation from deletion of nearby locations, randomization, discretization and subsampling, to mixing^30,31^. An extensive list of privacy protecting computation techniques has recently been published by Albouq *et al*.^32^.

The most relevant methods with regards to archaeological heritage sites are the discussions on obfuscating^33–35^ or cloaking of locations^36^. For the case that a precise location of a site is known but should not be revealed at the known accuracy level, three main options present itself in order to accomplish location obfuscation:

- The coordinates can be modified by randomizing the x and y component by a certain number^37^ or the numeric precision of the coordinate pair is decreased, e.g. instead of 8 decimal digits only 5 are displayed;
- The entire location is represented with a different shape such as a (randomized) buffer or shape around or off the center, or by a known administrative unit [cf.^20^];
- A nearby point of interest or other semantic representation of the location is given^38,39^.

While the above mentioned methods may be suitable for privacy preserving location based services and individuals as well as certain archaeological applications of limited scale, a problem arises when data sets of large geographic areas need to be obfuscated e.g. on a continental or global scale. For the first method, one needs to take into account that one decimal digit does not represent the same distance everywhere on the planet. Towards the poles the actual distance decreases. The obfuscation method is also directly linked to the respective data points. The second method carries with it a significant computational effort. Generating buffers or shapes is processing intensive and administrative units can vary substantially in size so that the obfuscation method becomes very inconsistent. For the third method, it might be difficult to choose a specific toponym from the available selection when too many are available and there might be areas where a suitable nearby point of interest is simply not available. Here we propose a globally scalable methodology to be applied as a procedure to obfuscate or cloak sites using a discrete global grid system (DGGS).

DGGSs are different from conventional geographic coordinate reference systems as they are designed to be an information grid, not a navigation grid. They divide the entire planet into a hierarchical tessellation of nested cells, providing a reference frame for the location of Earth observations and predictions. DGGSs provide a digital framework for geospatial information, quantizing or sampling conventional geospatial information into a discrete representation. The hierarchical structure allows for optimized integration, decomposition, and aggregation of data layers captured at different scales and resolutions. DGGS has its origin in the discourse about Digital Earth which approaches the creation of a three-dimensional digital twin of our globe in order to understand earth’s complex systems in a multidisciplinary manner integrating Big Geodata [cf.^40–42^]. DGGS is an approach on how to best represent the spherical earth in a geographic information systems and a number of approaches exist in how to unitize the earth’s surface^43^. We use a Discrete Global Grid System of hierarchical hexagonal cells as areal units developed by Sahr, White, and Kimerling,^43^. This approach is ideal for obfuscation due to its units having almost equal size across the entirety of the globe, allowing for very consistent obfuscation independent of location.

DGGSs are characterized by their tiling designs, cell address, quantization strategy, and associated mathematical functions. They provide discrete, nearly uniform fixed areas to assign values that describe the Earth. Spatial resolution is explicit as every item of information in a DGGS is associated with an area. The grid resolution can be designed to represent arbitrarily sized locations. Data stored as a multilevel global grid can be integrated without conflating between scales. Topology is explicitly defined within the relationship of cells and their values within a DGGS^43^. DGGS grid cells are relatively uniform, but also abstract, lending themselves as a tool to represent locations on the surface of the earth. The boundaries of a DGGS are standardized based on the information published by the Open Geospatial Consortium^44–46^ making DGGS reproducible.

A DGGS has a number of properties that make it a suitable mechanism for location obfuscation. The units of the grid system are abstract and not tied to specific anthropogenic features like administrative boundaries. The units are also entirely independent of the source data which needs to be obfuscated. Therefore, there is no way to reverse the obfuscation mechanism from the obfuscated data and obtain sensitive site locations information. The hexagons of our DGGS avoid semantic associations with nearby places or specific archaeological heritage sites. The DGGS provides a globally consistent standardized grid unlike local square grids which are subject to significant distortion when scaled. The DGGS is ISO/OGC standardized and the outcome will be the same grid no matter who generates the cells^44–46^. This increases interoperability and the potential for data integration. The grid’s hierarchical structure enables the obfuscation of archaeological heritage sites on different levels of accuracy which can be matched with different needs of stakeholders accessing the data.

## Data Records

Obfuscate.ipynb^47^:

10.6084/m9.figshare.25441735.v1

Subset of DGGS cells for coordinate obfuscation^48^:

10.6084/m9.figshare.25441795.v1

P3K14C dataset in DGGS cells of resolution 10 [Australia]^49^: 10.6084/m9.figshare.25441792.v1

P3K14C dataset in DGGS cells of resolution 12 [Great Britain]^50^: 10.6084/m9.figshare.25441780.v1

P3K14C dataset in DGGS cells of resolution 14 [Great Britain]^51^: 10.6084/m9.figshare.25441774.v1

P3K14C dataset in DGGS cells of resolution 8 [globally]^52^: 10.6084/m9.figshare.25441777.v1

## Data Availability

The authors declare that the data supporting the findings of this study are available within the paper or are else adequately referenced and publicly accessible.

## Code availability

The code is openly available under Obfuscate.ipynb: 10.6084/m9.figshare.25441735.v1. We use the following software libraries: Docker^53^, DGGRID^43^, PostgreSQL^54^, PostGIS^55^, PostgREST^56^ (for the API). We supply a working prototype through obfuscate.geoinsight.ai.
